# The Assessment of Chinese Children’s English Vocabulary—A Culturally Appropriate Receptive Vocabulary Test for Young Chinese Learners of English

**DOI:** 10.3389/fpsyg.2022.769415

**Published:** 2022-03-22

**Authors:** Laura E. de Ruiter, Peizhi Wen, Si Chen

**Affiliations:** ^1^Division of Human Communication, Development & Hearing, School of Health Sciences, The University of Manchester, Manchester, United Kingdom; ^2^Harvard Graduate School of Education, Cambridge, MA, United States; ^3^PACE Research Institute, Shenzhen, China

**Keywords:** English as a foreign language, receptive vocabulary, assessment, young language learners, China

## Abstract

Millions of Chinese children learn English at increasingly younger ages. Yet when it comes to measuring proficiency, educators, and researchers rely on assessments that have been developed for L1 learners and/or for different cultural contexts, or on non-validated, individually designed tests. We developed the Assessment of Chinese Children’s English Vocabulary test (ACCE-V) to address the need for a validated, culturally appropriate receptive vocabulary test, designed specifically for young Chinese learners. The items are drawn from current teaching materials used in China, and the depictions of people and objects are culturally appropriate. We evaluated the instrument’s reliability and validity in two field tests with a combined sample size of 1,092 children (181 children for the first field test and 911 children for the second field test, age range from 3.1 to 7.7, mean age: 5.2. Item Response Theory (IRT) analyses show that the ACCE-V is sufficiently sensitive to capture different proficiency levels and that it has good psychometric properties. ACCE-V scores were correlated with Peabody Picture Vocabulary Test scores, indicating concurrent validity. We found that children’s age and English learning experience can significantly predict the scores of ACCE-V, but the effect of English learning experience is greater. The ACCE-V thus offers an alternative to existing vocabulary tests. We argue that culturally appropriate assessments like the ACCE-V are fairer to learners and help promote an English learning and teaching environment that is less dominated by Western cultures and native speaker norms.

## Introduction

### Young English Learners in China

Over the past 20 years, English has gained importance as an academic subject in the Chinese education system. In 2001, the Ministry of Education (MOE) of China launched the “Guideline for Promoting English Teaching in Elementary Schools,” which made English a compulsory subject starting from the third (rural areas) or the first grade (urban areas) of elementary school (Ministry of Education of the People’s Republic of China, 2001). In the Chinese College Entrance Examination (Gao Kao), English is weighted as much as Chinese and mathematics.

Because of the importance of English in children’s academic achievement, many parents try to boost their children’s English skills by sending them to after-school and cram schools (Feng, 2012). They also believe that the best way to achieve good English proficiency is to start learning English at an early age, that is, before they enter elementary school and receive English instructions as part of the regular curriculum. As a consequence, more children at increasingly younger ages are learning English as a foreign language (EFL) (Hu and McKay, 2012). In 2016, more than 210 million young EFL learners under the age of six were taking English courses in more than 50,000 private English institutes in mainland China (Sun et al., 2016), and the numbers have almost certainly only increased since then. Recently, China has seen a significant growth in online English tutoring. A report showed that more than 26.9% users of all the K-12 (kindergarten through 12th grade) online English teaching courses in China are aged 3–6 years (Aurora Mobile, 2020).

In China, public kindergartens are prohibited from teaching English (Ministry of Education of the People’s Republic of China, 2018). Private kindergartens, in contrast, are allowed to teach English (Chen et al., 2020). Although China has recently issued a series of policies that prohibit online classes for children, including English (Ministry of Education of the People’s Republic of China, 2021), many parents are still enthusiastic about having their young children learn English. Some kindergartens even hire foreign teachers to carry out immersive English teaching. Although private schools charge much higher fees than public schools, they are still popular with parents, especially with those with higher socio-economic status (SES). Since the government introduced restrictions on early English teaching in 2021 (Ministry of Education of the People’s Republic of China, 2021), English is being learned by Chinese preschoolers exclusively in private kindergartens.

### Challenges for Early English Teaching in China

Early English teaching in China faces several challenges with respect to quality. First, there are no educational standards for English teaching in kindergartens. Individual kindergartens or even individual teachers determine the content of teaching. High-quality kindergartens may decide to have courses and class plans reviewed and checked by specialized teaching and research departments of their local Ministry of Education. However, this kind of quality control is entirely voluntary, and in many kindergartens, there is no quality control whatsoever. This problem is compounded by the fact that foreign teachers in Chinese kindergartens are generally highly mobile, and that kindergartens have a high turnover rate. These constant changes in teaching personnel make it difficult to deliver high-quality education in a consistent manner.

Second, children in Chinese kindergartens are automatically grouped by age rather than proficiency level. However, unlike with Mandarin, age is not necessarily a good indicator of proficiency level in English, because children come to class with different backgrounds, due to the fact that many parents are pursuing different strategies to have their children learn English, ranging from apps to private tutors. This age-based grouping can make it difficult to cater to the needs of children with different levels of English proficiency.

In this environment, it is very difficult for teachers to provide differentiated content to the children that is adequate for their proficiency level. Very experienced teachers may be able to gauge children’s English proficiency through regular interaction and observation. However, given that most teachers are inexperienced teachers and are often teaching the children only for a relatively short period, it is not easy for them to assess the actual proficiency level of individual children. Under these circumstances, an appropriate English proficiency test can provide this information. However, currently, there is a lack of assessment tools suitable for young Chinese children.

### Currently Available Tests

#### L2-Specific Tests

There are currently three sets of tests aimed at young learners of English that are also available in China: the Cambridge Young Learners English Test (CYLE), developed by Cambridge ESOL Examinations, the Pearson Test of English Young Learners (PTE Young Learners), developed by Pearson, and the TOEFL Primary test, developed by ETS.

The CYLE test series tests how well 7–12-year–olds are performing in the skills of listening, speaking, reading, and writing. It has so far been administered in 68 countries and has more than 360,000 test takers annually (Chik and Besser, 2011). The PTE Young Learners, called Starters is aimed at learners between 6 and 13 years, also assessing the four language skills. TOEFL Primary is for learners 8 years and up and assesses reading and writing and speaking. All three tests are administered at designated test centers, with TOEFL Primary being the only one that also offers institutional testing at schools.

Although these three tests are aimed at children above kindergarten age (3–6 years in China), they are nonetheless often used in Chinese kindergartens. This is highly problematic for several reasons. A major feature of these tests is that their format requires that children can already read and write, which is not the case for most kindergarten children in China who are just beginning to learn English. When used with preliterate children, these tests will therefore produce inaccurate results—a child may have a certain level of oral proficiency, but this will not be captured by a test that requires literacy. Furthermore, these tests are designed for children above elementary school and lack the play-based interface that is necessary to engage young children. And finally, the tests are designed according to the cultural norms of Western countries (specifically the United States, the United Kingdom, Canada, and Australia), which makes them less accessible for Chinese children who lack the cultural background knowledge, as we will discuss in more detail in the next section.

Another issue with these tests is that they are designed to cover all areas of language ability, with a focus on phonological awareness and grammar. However, the consensus in early language teaching is that for very young children, teaching should be focused on developing lexical competence and no explicit grammar instruction, as children do not develop the necessary metalinguistic skills until much later (Curtain and Dahlberg, 2010; Shin and Crandall, 2014). In line with this, any instrument for assessing young learners’ proficiency should focus on vocabulary.

#### Vocabulary Tests

When it comes to measuring the vocabulary of young Chinese EFL learners, researchers and teachers typically rely on assessments such as the PPVT (Dunn and Dunn, 2007) or the British Picture Vocabulary Scale, BPVS (Dunn et al., 2009). These tests have been developed over many years, and typically have very good psychometric properties. However, they are aimed at children learning English as their first language (L1). Using these tests for EFL learners is problematic for two different but related reasons.

The first reason is that the learning environment for children who learn English in an educational setting differs from that of children learning it in their home environment. Children who initially acquire words in their home environment learn these from conversations with and among adults as well as through activities such as pretend to play and book reading (Ninio, 1983; Snow et al., 1991). Their early vocabulary is characterized by words that reflect what is relevant for the child, such as family members (e.g., *mommy, daddy*), toys (e.g., *ball, teddy*), body parts (e.g., *toe, hand*), or food items (e.g., *sandwich, cookie*). In line with this, the structure of vocabulary tests like the PPVT is based on the idea that in the child’s experience, some words are more frequent than others, and that children will acquire more frequent words before less frequent words (Dunn and Dunn, 2007).

However, the words used in English lessons in Chinese schools are different from what a child may encounter when it learns English from parents and others at home. English lessons often introduce school-related vocabulary (e.g., *classroom, pencil, ruler*), places (e.g., *library, hospital, post office*), shapes and colors (e.g., *rectangle, red*), and animals (e.g., *elephant, snake*). Thus, testing Chinese young EFL learners with words that children growing up in an English-speaking environment typically learn at home will not provide an accurate picture of their vocabulary knowledge.

The second, related reason why these tests are problematic is that the items are based on the dominant culture of the country for which the test was developed (e.g., United States, United Kingdom). This applies both to the types of items used as well as the way items are depicted. An example for the first issue items (‘items’ being used here to mean both targets and distractors) like *muffin* and *pretzel* (both used in the PPVT), items that are unlikely to be known to young Chinese children. Examples of culturally specific depictions are a traditional English teacup with a handle on a saucer (used in the BPVS), which are not common in China, or a castle in European medieval style (used in the PPVT), which is very different from the way castles look in China.

Recent research found the PPVT to be less reliable for L2 learners with limited English experience and proficiency. Wood et al. (2015) tested both Spanish-speaking kindergarteners’ and monolingual kindergarteners’ vocabulary using the PPVT-4. They observed that the relationship between the difficulty level of the items in the PPVT (which is indicated by the order of items in the test) was positively related to children’s error scores in both groups (i.e., children made more errors with more difficult items). However, this relation was much stronger in English monolingual children than in Spanish L2 learners of English. In other words, the findings suggest that the difficulty assumptions that the test is based on do not hold to the same extent for L2 learners as they do for L1 learners. Goriot et al. (2018) administered the PPVT-4 to pupils in the Netherlands in six different age groups (4–15 years old). They found that the test had low reliability scores (as measured by Cronbach’s alpha) for learners with limited proficiency; these were predominantly children in the youngest age of 4–5 years. They also found an effect of the children’s L1. English words that had cognates in Dutch (e.g., *penguin* and *pinguin*) tended to be easier for participants than words that did not, most likely because participants were able to guess the meaning. Like Wood and Pena’s study, this study showed that the test’s reliability is lower with L2 speakers. In addition, the participants in Goriot et al. (2018) had the advantage of speaking a typologically close L1 and of being culturally closer to America. Potential issues with tests like the PPVT are arguably more pronounced in young Chinese learners of English, whose L1 does not have any linguistic similarities with English and who have less experience with Western culture.

The learning environment of young Chinese English learners is different also from that of dual language learners in an English-speaking country (e.g., Hispanic English language learners in the United States, who speak Spanish at home, but who learn and speak English at school, or EAL learners in the United Kingdom who speak Pashtu at home but speak English at school). Children whose home language is not English have less exposure to English than children whose home language is English (e.g., Cote and Bornstein, 2014), and they score consistently lower on English vocabulary tests than their monolingual counterparts (REF). But unlike foreign language learners in China, these children are immersed in English continuously at school (not only during English lessons), and are likely to interact with others (peers, teachers) in English on a regular basis. Researchers who work with this population have pointed out that test validity is threatened when available norms are based on monolingual children, when the child’s cultural experiences do not match test expectations, or when the items are not presented in a way that allows the child to demonstrate competence (Peña and Halle, 2011). This problem is exacerbated for children who do not even have minimal experience with the cultural norms reflected in test items, such as young Chinese learners of English living in China.

Against this background, we developed a new vocabulary test, designed specifically for young Chinese learners of English. But before we move on to describing this new instrument, we briefly want to discuss what it means for a young learner to “know a word” (in comprehension).

### “Knowing a Word”

To know a word usually means that someone knows its basic meaning (denotation). For L1 speakers or more advanced learners, one would assume that they also have an understanding of evaluative meanings (connotation), an understanding of its grammatical form, an awareness that the word can have multiple meanings (e.g., *to run across the field* vs. *to run a business* vs. *he had a good run*), or knowledge of which register a word belongs to (e.g., formal, casual).

In the case of young learners, we assume knowledge only of its denotation for the word’s most frequent use, for example, understanding that run means “move using your feet/limbs at a speed faster than a walk.” Traditionally, vocabulary tests use word families as their unit of recognition (Schmitt, 2010). A word family comprises the base word and its inflections and most common derivations. For example, the words *run*, *runs*, *ran*, *running*, and *runner* would all be assumed to be of the same word family. In other words, if a learner knows a word such as *run*, it is assumed that they will also know the meaning of *runner*, or at least be in a position to guess its meaning. However, some studies have challenged this assumption, finding that learners may in fact not know the other family members (Nation, 2006). Ward and Chuenjundaeng (2009), for example, conducted a study with Thai EFL learners, focusing on their suffix knowledge. They conclude that their findings “contradict the assumption that knowledge of headwords implies knowledge of word families, at least with lower-level students from non-Latinate L1 [first language] backgrounds” (p. 465). For this reason, we refrain from making assumptions regarding the size of a learner’s lexicon.

### The Current Study: Purpose and Use of the Assessment of Chinese Children’s English Vocabulary

As we discussed, existing vocabulary tests are not well suited for young Chinese learners of English. Both educators and researchers would benefit from a receptive vocabulary test that is specifically designed for this growing population. For educators, a suitable assessment tool will help understand the level of children’s English development accurately, and this information can help educators set English learning goals and design curriculum content suitable for children’s developmental level. For researchers, assessment tools are also needed to estimate children’s English ability, for example in the context of evaluating the effects of educational experiments and intervention projects. We (a group of early childhood education, psychology, and psycholinguistic researchers) therefore developed the Assessment of Chinese Children’s English—Vocabulary (ACCE-V). The test was commissioned by the PACE Research Institute, which focuses on research on early childhood education in China.

The ACCE-V is a multiple-choice, receptive vocabulary test for young Chinese (Cantonese- and Mandarin-speaking) learners of English between 4 and 7 years and assesses vocabulary knowledge that is relevant in the context of Chinese primary English education. Because it does not require reading, can be used with preliterate children. Since the purpose of the test in educational settings is to provide teachers with information about the children’s proficiency to allow them to tailor their teaching accordingly, scores are meant only for educators and will not be communicated to parents. Educators will receive standardized scores that interpret children’s scores based on the group means and standard deviations. By avoiding communicating the scores to parents, we believe that the ACCE-V will not add to the existing ‘testing culture’ in China.

The current study describes the design and validation of the ACCE-V. We ask two research questions:

(1) Does the ACCE-V have acceptable psychometric characteristics?

(2) What is the relationship between the ACCE-V, children’s demographic features (age and gender), and children’s English learning experience?

Regarding question (2), we hypothesize that there is little correlation between children’s age and their vocabulary scores, while the correlation between children’s English learning experience and vocabulary scores is greater.

## Materials and Methods

### Participants

#### First Field Test Participants

One-hundred-and-eighty-one children between 3 and 7 years of age (*M* = 5;04^1^) were recruited from two preschools in two major cities in China (see Table 1): one in eastern China (*n* = 72) and one in southern China (*n* = 109). Socio-economic background is often correlated with educational outcomes. We therefore wanted to include some information on the participants’ SES. As it was not possible to collect information about parents’ income or their educational background, we used the tuition costs of the preschools and publicly available economic information about the catchment areas of the preschools as indicators (here: housing prices). The first preschool served predominantly middle SES families: Its tuition was 28% higher than the average tuition in the city, while the average housing price was ¥ 45,280/m^2^, which is slightly lower than the average housing price of both the cities (which is ¥ 50,000/m^2^ in both cities). The second preschool served predominantly high SES families, as evidenced by the fact that its tuition is ten times the average tuition in the southern China city and the housing price in its catchment area is more than two times the average (¥ 108,920/m^2^). Based on the total number of children enrolled in each preschool, 40% of all children were randomly selected from each grade. Of the 181 children in our sample, 75 were female and 106 were male.

**TABLE 1:** Descriptive statistics.

Field test	*N* (%)	Mean	Standard deviation	Standard error of the mean	Skewness	Kurtosis	95% CI
First field test	181						
Gender (female)	75 (41.43%)						
Children’s age	181 (100.00%)	5.30	0.69	0.05	–0.24	2.42	5.20-5.40
**SES (housing price, thousand per square meter in RMB)**							
Middle SES (Southern China)	109 (60.22%)	45.28	8.10	1.81	0.15	2.26	41.49–49.07
High SES (Eastern China)	72 (39.78%)	108.92	22.10	6.38	0.06	1.54	94.87–122.96
Second field test	911						
Gender (female)	405 (44.46%)						
Children’s age	911 (100.00%)	5.12	0.96	0.03	–0.10	2.06	5.06–5.19
**SES (housing price, thousand per square meter in RMB)**							
Low SES (Southern China)	199 (21.84%)	26.50	11.49	3.19	–0.02	1.51	19.56–33.45
Middle SES (Southern China)	112 (12.29%)	54.58	3.85	1.28	–0.35	1.85	51.62–57.54
Middle SES (Eastern China)	130 (14.27%)	77.93	6.16	2.05	0.30	2.01	73.19–82.66
Middle-to-high SES (Southern China)	383 (42.04%)	93.41	25.80	6.66	–2.04	8.18	79.12–107.70
High SES (Eastern China)	87 (9.54%)	108.92	22.10	6.38	0.06	1.54	94.87–122.96

#### Second Field Test Participants

Nine-hundred-and-eleven children participated in the second field test. The children were randomly selected from each grade in each preschool. Of these, 405 were female and 506 were male (mean age = 5;01).

We employed the same selection criteria as in the first field test and recruited six kindergartens and one elementary school for the second field test. Four kindergartens were located in a metropolis in southern China (*N* = 694), one from a low SES neighborhood with tuition 36% lower than the average school tuition in the city, and with an average housing price of ¥ 26,500/m^2^; two from middle SES neighborhoods with tuition 28% higher than the average tuition and with an average housing price of ¥ 54,580/m^2^, and one from a middle-to-high SES neighborhood with tuition 28% higher than the average tuition and with an average housing price of ¥ 93,410/m^2^. The elementary school (with tuition 28% higher than the average tuition in the city) was from a middle-to-high SES neighborhood. Two kindergartens were in a metropolis in eastern China (*N* = 217; one from a middle SES neighborhood with tuition six times the average tuition and with an average housing price of ¥ 77,930/m^2^ and one from a high SES neighborhood with tuition ten times the average tuition and with an average housing price of ¥ 108,920/m^2^).

Some children did not attend preschool on the second day of testing, or they did not want to complete one or several of the tests, often because they were shy. Of the children in the first cohort (*N* = 558), 22 children did not complete either of the two forms of the ACCE-V, and 21 of these did also not complete the PPVT-4. One child completed only Form A, but none of the other tests. Of the children in the second cohort (*N* = 353), 12 did not complete either of the two forms of the ACCE-V, and of these twelve, four did not complete these tests on the re-test date, either. One child did not complete Form B on the first day but did complete Form A and both forms on the re-test date.

### Test Construction and Item Development

#### Target Item Selection

**FIGURE 1:**
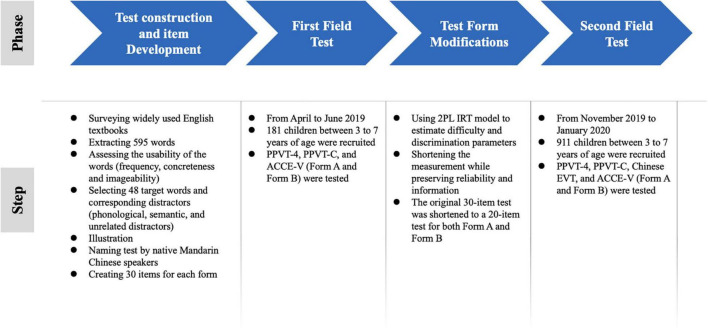
Procedure of the ACCE-V development.

The main purpose of the ACCE-V is to assess the level of receptive vocabulary knowledge relevant in the context of Chinese primary English education. Our search for target items therefore began by surveying the most widely used English textbooks and working books developed for first and second graders in China (see Table 2). From these books, we extracted the English words used in exercises, texts, and instructions (excluding pronouns and conjunctions). Altogether, 595 words (nouns, pronouns, adjectives, adverbs, verbs, and prepositions) were extracted. The procedure of the ACCE-V development was illustrated in Figure 1.

**TABLE 2:** Titles and grades of the English text- and workbooks used in the item development of the ACCE-V.

Book title English	Book title Chinese	Grade level
English Textbook—Starting Line (First Grade First Semester)	义务教育课程标准实验教科书：英语新起点（一年级上册）	Grade 1
English Textbook—Starting Line (First Grade Second Semester)	义务教育课程标准实验教科书：英语新起点（一年级下册）	Grade 1
English Textbook—Starting Line (Second Grade First Semester)	义务教育课程标准实验教科书：英语新起点（二年级上册）	Grade 2
English Textbook—Starting Line (Second Grade Second Semester)	义务教育课程标准实验教科书：英语新起点（二年级下册）	Grade 2
English Reading Comprehension Series (First Grade)	全新英语阅读（一年级阅读理解）华东师范大学出版社	Grade 1
English Reading Comprehension Series (Second Grade)	全新英语阅读（二年级阅读理解）华东师范大学出版社	Grade 2
English Listening Series (First Grade)	全新英语听力（一年级提高版）华东师范大学出版社	Grade 1
English Listening Series (Second Grade)	全新英语听力（二年级提高版）华东师范大学出版社	Grade 2
English Oral Communication Workbook (First Grade First Semester)	口语交际英语活动手册（一年级上册）	Grade 1
English Oral Communication Workbook (First Grade Second Semester)	口语交际英语活动手册（一年级下册）	Grade 1
English Oral Communication Workbook (Second Grade First Semester)	口语交际英语活动手册（二年级上册）	Grade 2
English Oral Communication Workbook (Second Grade Second Semester)	口语交际英语活动手册（二年级下册）	Grade 2
One Lesson One Practice (English): First Grade First Semester	华东师大版：一课一练一年级英语（第一学期）	Grade 1
One Lesson One Practice (English): First Grade Second Semester	华东师大版：一课一练一年级英语（第二学期）	Grade 1
One Lesson One Practice (English): Second Grade First Semester	华东师大版：一课一练二年级英语（第一学期）	Grade 2
One Lesson One Practice (English): Second Grade Second Semester	华东师大版：一课一练二年级英语（第二学期）	Grade 2
New Concept: First Things First!	新概念英语：英语初阶	Grade 1

Each word was assigned to one of 18 content categories (actions, animals, body parts, attributes^2^, people, buildings and spaces, vehicles, household objects, clothing and accessories, shapes and colors, nature and landscapes, food, plants and fruit, books and money, toys and recreation, times, numbers, prepositions, pronouns, abstract concepts). We assessed the usability of the words based on frequency measures, concreteness, and imageability.

A word’s frequency is measured by counting how often it occurs in a corpus (e.g., in a collection of books or newspapers). We included frequency measures as a criterion because it seems fair to assume that more frequent words will more likely be found in different textbooks (and consequently be used in more English language classes and known to more children), whereas less frequent words reflect more idiosyncratic choices by a textbook publisher.

In the absence of a corpus of words used in Chinese English-language classes, we used two widely used databases: the CELEX database (Baayen et al., 1996), which is based on the COBUILD corpus with around 17.9 million tokens (from both written and spoken sources), and the SubtlexUS database (Brysbaert and New, 2009), which contains 50 million tokens and is based on American movies and TV series subtitles. We used the Cob frequency (from CELEX) and the SUBTLwf (from SubtlexUS) to identify and exclude low-frequency words (e.g., *narrator*, *magnificent*), and to decide between semantically similar words (e.g., *lollipop* and *candy*), assuming that more frequent words were more likely to be taught (in this case *candy* has a higher frequency than *lollipop*).

Concreteness and imageability were used to restrict the pool of possible target words to those that would be more likely to be known to children and that could be depicted clearly using drawings. Imageability is defined as the ease with which a word gives rise to a sensory mental image (Paivio et al., 1968), while concreteness refers to the ability to see, hear, and touch something. Empirically, words like *difference* or *against* tend to get lower concreteness ratings than words like *banana or running*.^3^ Not surprisingly, words with lower concreteness ratings are also typically more difficult to illustrate. We gauged a words’ imageability and concreteness using our own and the illustrator’s (see below) introspection and our experience with creating visual stimuli for young children.

#### Test Construction

##### Distractors

We then selected a subset of 48 words as potential target words. For each word, three distractor words were selected: a phonological distractor, a semantic distractor, and an unrelated distractor. The phonological distractors share the initial phoneme or onset with the target (e.g., *skirt* and *square*). The semantic distractors were from the same content category, and semantically related for instance through being a subordinate of the same superordinates the target word (e.g., *eye* and *nose* being subordinates of *face*), or being the opposite of the target word (e.g., *losing* and *finding*). Unrelated distractors were neither semantically nor phonologically related. The distractors were always of the same part of speech as the target word. Where possible, distractors were selected that had a similar frequency as the target word. Distractors were also selected to be concrete and to have high imageability.

##### Illustration

**FIGURE 2:**
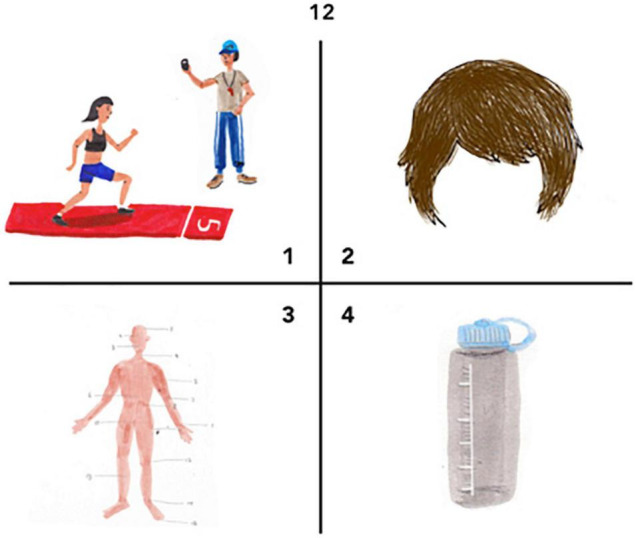
Example of an item from ACCE-V. The target word is body. The body is depicted using a traditional schematization of the body as often used in the context of Traditional Chinese Medicine.

A professional illustrator created color pictures for all target and distractor words. The illustrator was a Chinese–English bilingual and born and raised in China who knows the living environment of typical Chinese children. This is important, as a main goal of ACCE-V is to be culturally appropriate both in terms of the items used and in terms of the illustrations. In other words, the illustrations should be in a style that is familiar to Chinese children. An example is provided in Figure 2.

We tested whether the illustrations would evoke the intended concepts in Chinese native speakers using a picture naming paradigm: Each picture was presented to 14 native speakers of Mandarin Chinese (four adults, ten children between 5 and 7 years; eight females, six males) living in China, and they were asked to name what was shown on each picture in Mandarin. The overall agreement was 74.6%. Modifications were made for those pictures that showed low naming agreement (less than 50%) or high agreement but not on the intended (Mandarin translation of the English) word (e.g., most participants named the picture intended to show *face* as *head*, which led to removing the hair). Modified pictures were tested again to ensure that they had more than 80% agreement.

##### Test Forms

In a next step, we selected 30 target words and their distractors. In addition, we also created a second form of the test, in which some of the semantic distractors of Form A served as targets, and the targets of Form B served as semantic distractors. The rationale for having two different but equivalent forms is that it allows re-testing without practice effects.

### Measures

#### Criterion Validity

To compare ACCE and other vocabulary tests, we chose the Chinese version of PPVT (Lu and Liu, 1998, henceforth: PPVT-C) and the English version of PPVT (Dunn and Dunn, 2007, the PPVT-4).

The PPVT-C contained 115 items with a possible score ranging from 0 to 115. It was translated and validated in Taiwan. Like the ACCE, the PPVT-C is a forced-choice picture selection format, in which the child is presented with a word and then asked to select the target picture matching that word from an array of four pictures. If a child answers five out of seven consecutive items wrong, the examiner would stop the test and record the score. The internal consistency of the PPVT-C was 0.83, and that of the PPVT-4 was 0.89.

In addition to the revised ACCE-V vocabulary test (form A and form B) and PPVT-C (Lu and Liu, 1998), we also included the English PPVT-4 (Dunn and Dunn, 2007) and the Chinese Expressive Vocabulary Test (EVT) to test criterion validity (concurrent validity). The EVT in Chinese was adapted by the Child Language Research Center (CLRC) at East China Normal University (see Chen et al., 2018 for details). Possible scores range from 0 to 124. The internal consistencies (Cronbach’s alpha) of the PPVT-C, the English PPVT-4, and the Chinese EVT in our sample were 0.82, 0.96, and 0.73, respectively.

#### Demographic Information

**Children’s age** was calculated by subtracting children’s birth date from the test date, counted in months.
**Children’s gender** was a binomial variable with 1 for girl and 0 for boy.
**Foreign teacher** was a binomial variable, representing whether there was a foreign teacher in children’s classroom (1 = yes, 0 = no).
**Housing price** was the average price in the catchment area of the preschool, in Yuan per square meter.
**Tuition** was the tuition of children’s preschool, counted in Yuan per month.

### Analytic Approach

#### RQ1: Assessment of Chinese Children’s English Test Reliability and Validity

To assess the psychometric properties of the ACCE-V, we used both Item Response Theory (IRT) and classical test theory to determine the test’s reliability and validity. After the first field test, we modified or removed items that did not have satisfactory properties, and then tested the modified version again in a second field test. We used a two-parameter (2PL) IRT model to fit the data.

#### RQ2: Assessment of Chinese Children’s English and Children’s Demographic

Once we had established that the ACCE had good psychometric properties, we used multiple regression models to compare the children’s age, gender, and English vocabulary scores. In the regression models, the outcome (English vocabulary score) was modeled as a linear combination of the predictor variable (age), controlling for gender, foreign teacher, housing price, and tuition.

Analyses were conducted using STATA 15.0 (StataCorp, 2017) and R 3.6.2 (R Core Team, 2019).

### Procedure

The first data collection took place between April and June 2019. Ten Chinese-English bilingual research assistants working in the preschool (with at least a bachelor’s degree) were selected and trained by the authors. The training covered the administration of the test, including training the pronunciation of each target word. The purpose of this study, testing procedure, potential risk, and privacy were sent to the preschool administrators. The preschool administrators informed each parent through the parent committee who collected parents’ assent to the testing. Of the children whose parents agreed, children who also agreed were tested. All children were told that it is fine to stop the test at any time.

Children were tested individually by research assistants in their kindergartens. Of the 181 children, 143 completed both forms to gauge alternate form reliability. The two forms were administered in random order to counter-balance the test order. In addition, these 143 children completed the PPVT-C, and 34 completed the PPVT-4. The PPVT-C was administered before the ACCE-V test, and the PPVT-4 at the end. The administration of one form of the ACCE-V test took about 10 min. The administration of the PPVT-C and the PPVT-4 varied depending on the children’s vocabulary level between 5 and 15 min. There was a short break between each test.

The same group of assistant researchers conducted the second field test. All children completed all tests in 2 days. From November 2019 to January 2020, 558 were tested on the PPVT-C and Chinese EVT on the first day and on both forms of the ACCE-V (order counter-balanced) and English PPVT-4 on the second day. In November and December 2020, the other 353 children were tested on both forms of the ACCE-V on 1 day, and then again on both forms 1 week after (order counter-balanced) to gauge the instrument’s test–retest reliability.

## Results

### RQ1: Assessment of Chinese Children’s English Test Reliability and Validity

Table 3 shows the correlations and descriptive statistics for the ACCE-V Basic Form A and Form B, and for the PPVT-4 and PPVT-C. As can be seen in Table 3, both forms had medium to high correlations with the PPVT, and medium correlations with the PPVT-C. In addition, scores in both forms were highly correlated with each other.

**TABLE 3:** Correlations and descriptive statistics for the four tests (ACCE-V Form A, ACCE-V Form B, PPVT-4, and PPVT-C).

	1	2	3	4
(1) ACCE-V Form A	–			
(2) ACCE-V Form B	0.909***	–		
(3) English PPVT	0.622***	0.491***	–	
(4) PPVT-C	0.331**	0.43*	0.462***	–
Max. achievable score	30	30	228	115
Mean	15.56	13.92	42.35	50.93
Median	15	12	42	47.5
Standard deviation	7.05	7.14	10.3	19.14
Standard error of the mean	0.54	0.58	1.27	1.41
Skewness	0.15	0.43	–0.12	0.63
Kurtosis	1.85	2.15	2.42	3.30
95% CI	14.59–16.72	12.77–15.05	39.53–44.59	48.15–53.71

N = 181. *p < 0.05; **p < 0.01; ***p < 0.001.

#### Test Form Modifications After the First Field Test

**FIGURE 3:**
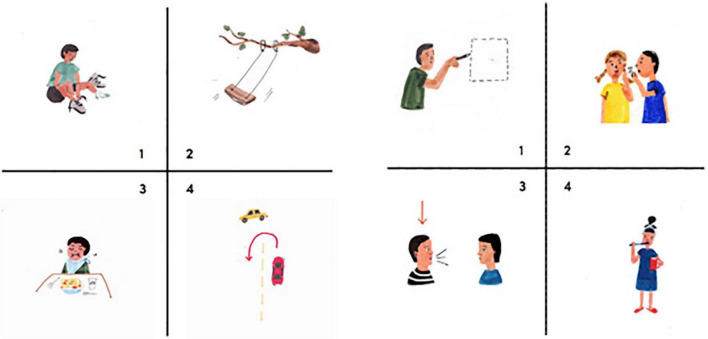
Examples of removed items due to low discrimination indices. The target for the **left panel** was to turn (option 4), the target for the **right panel** was to talk (option 3).

The uni-dimensionality assumption of the data was evaluated by using a principal components analysis (PCA). For Form A, the PCA showed that the first component (26.89%) accounted for substantially more variation than the second component (7.00%) and subsequent composites, indicating that Form A was measuring a unidimensional ability. Results were similar for Form B, where the first component accounted for 28.43% of the total variance whereas the second component accounted for only 7.72%. The uni-dimensionality assumption was thus met. We used a 2PL model to fit the data, allowing us to estimate difficulty and discrimination parameters for each item. Items with lower discrimination parameter estimates and items that were too easy were removed. Examples were illustrated in Figure 3.

**FIGURE 4:**
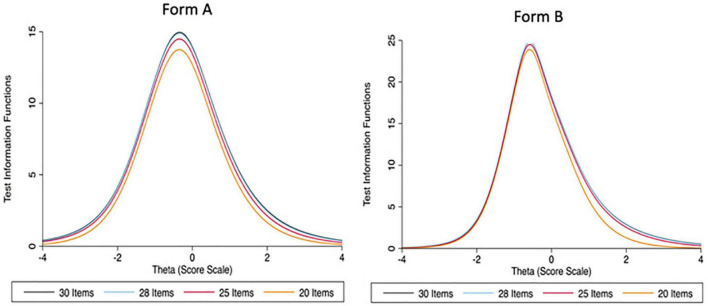
Test Information Functions (TIF) for Form A and Form B of the first version of the ACCE-V for 30 items, 28 items, 25 items, and 20 items, respectively.

To shorten the measurement while preserving the reliability and information, the original 30-item test was shortened to a 20-item test by using the Test Information Function (TIF) and Conditional Standard Error of Measurement (CSEM). Both measures allowed us to compare the total information provided by a different shortened version of a test (see Figure 4). For each form in the field test, we selected 20 items out of 30 were that minimized the CSEM and maximized the TIF. In addition, difficulty parameter estimates were used to arrange items from easy to difficult in each form.

#### Internal Consistency

Internal consistency measures were calculated for each 6-month age band. Table 4 shows the internal consistency measures (split-half reliability, Cronbach’s α, and standard error of measurement) for each of the age bands. All measures indicate high to very high internal consistency of the ACCE-V, indicating that it is suitable for all age ranges.

**TABLE 4:** Split-half reliability, Cronbach’s **α**, standard error of measurement, and alternate form reliability for each 6-month age band.

Age	*N*	Split-half reliability		Cronbach’s alpha		Standard error of measurement		Form A		Form B		Mean difference	*r*
		Form A	Form B	Form A	Form B	Form A	Form B	Mean	*SD*	Mean	*SD*		
3.0–3.5	36	0.73	0.79	0.82	0.81	1.4	1.45	7.78	4.3	8.08	4.21	–0.3	0.65
3.6–3.11	109	0.78	0.8	0.85	0.85	1.42	1.35	7.83	4.6	8.61	4.66	–0.78	0.82
4.0–4.5	107	0.85	0.83	0.87	0.87	1.26	1.37	9	4.96	9.82	4.96	–0.82	0.86
4.6–4.11	124	0.86	0.78	0.89	0.87	1.29	1.69	9.28	5.27	10.26	4.97	–0.98	0.87
5.0–5.5	152	0.89	0.87	0.9	0.88	1.28	1.23	10.33	5.51	11.1	5.13	–0.77	0.88
5.6–5.11	163	0.83	0.84	0.89	0.87	1.32	1.27	9.14	5.31	9.55	5.16	–0.41	0.9
6.0–6.5	119	0.84	0.83	0.88	0.88	1.29	1.27	8.49	5.2	9.21	5.16	–0.72	0.89
6.6–6.11	37	0.82	0.89	0.91	0.9	1.45	1.4	9.85	5.97	10.31	5.89	–0.46	0.93

*N = 911.*

#### Test–Retest Reliability

Both forms of the ACCE-V were completed twice by 340 children, with a test interval of 1 week. The correlation for Form A was *r* = 0.86 and *r* = 0.85 for Form B, indicating good test–retest reliability.

#### Alternate Form Reliability

We determined alternate form reliability by correlating scores from Form A and Form B. Overall, the alternate form reliability was 0.85. Table 4 shows the correlations for each individual age band. There were moderate to strong positive correlations between both forms, indicating good alternate form reliability. At *r* = 0.65 the correlation is lowest in the youngest age band (3.0–3.5 years), and the only band with a correlation below 0.8. We believe that the youngest age group may have had more difficulty keeping their concentration throughout the multiple assessments, despite the pauses in between. In addition, this age band had the smallest sample size (*N* = 36).

#### Concurrent Validity

The ACCE-V scores were correlated with the PPVT-4, the PPVT-C, and the Chinese EVT. Table 5 shows the correlation of the test scores with each other.

**TABLE 5:** Correlations and descriptive statistics for the five tests (ACCE-V Form A, ACCE-V Form B, PPVT-4, and PPVT-C).

	1	2	3	4	5
(1) ACCE-V Form A	–				
(2) ACCE-V Form B	0.879***	–			
(3) English PPVT-4	0.808***	0.802***	–		
(4) Chinese PPVT	0.396***	0.380***	0.341***	–	
(5) Chinese EVT	0.346***	0.333***	0.297***	0.615***	–
Max. achievable score	20	20	228	115	124
Mean	9.09	9.78	25.16	40.44	56.84
Median	8.00	9.00	22.00	36.00	58.00
Standard deviation	5.25	5.12	14.95	20.25	14.92
Standard error of the mean	0.18	0.17	0.65	0.86	0.72
Skewness	0.33	0.34	0.47	0.61	–0.38
Kurtosis	2.00	2.11	2.22	2.79	3.33
95% CI	8.75–9.44	9.44–10.12	23.89–26.43	38.75–42.12	55.43–58.26

N = 911. ***p < 0.001.

ACCE-V scores were most strongly correlated with PPVT-4 scores. Since the PPVT and the ACCE-V both assess children’s English receptive vocabulary, this is to be expected and desirable. Correlations with the PPVT-C are moderate, showing that children’s Mandarin skills and their L2 English skills are related. The strength of the correlation is in line with what is generally found for receptive L1 and L2 vocabulary in young learners (Atwill et al., 2007; Karlsen et al., 2017; Grøver et al., 2018). The correlations with the Chinese Expressive Vocabulary Test are lowest at around 0.3. This is not surprising, given that both tests are measuring proficiency in different languages (English vs. Mandarin) and in different modalities (receptive vs. productive).

#### Item Analysis

**FIGURE 5:**
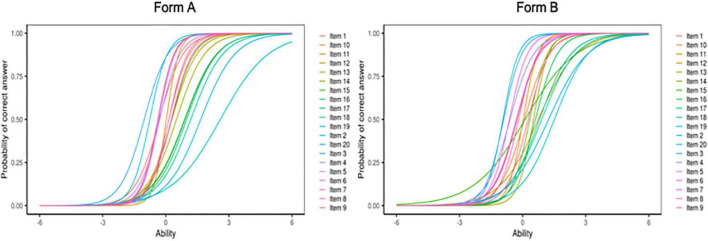
Item Characteristic Curves for Form A and Form B of the ACCE-V. Plot created with Shiny Item Analysis.

We again used a 2PL model to measure the items’ difficulty and discrimination. All items had very good discrimination indices and provided a good range of difficulty indices (see Table 6; Figure 5). The most difficult items tended to be verbs (e.g., *putting*) and prepositions (e.g., *on*).

**TABLE 6:** Range, mean, and standard deviation of the discrimination and difficulty indices of the ACCE-V Form A and Form B.

	Discrimination			Difficulty		
	Range	Mean	*SD*	Range	Mean	*SD*
Form A	0.99–3.23	2.04	0.58	–1.66 to 2.32	–0.02	0.93
Form B	0.94–2.87	1.91	0.55	–1.54 to 1.28	–0.18	0.78

**FIGURE 6:**
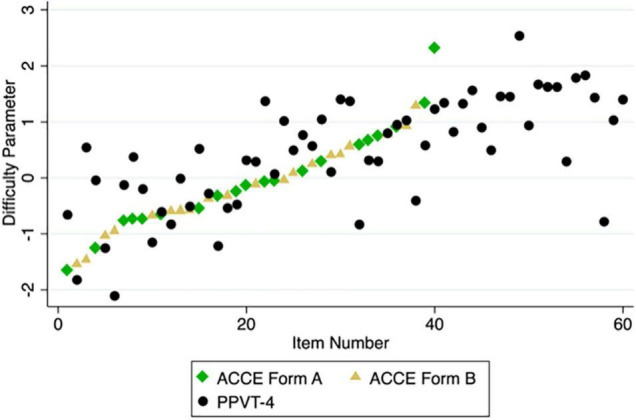
Difficulty parameters of items in the ACCE-V Form A, ACCE-V Form B, and the PPVT-4 in the order in which the items are presented.

We also compared the ACCE-V’s difficulty parameters with those of the PPVT-4. As can be seen in Figure 6, some items from the PPVT that are presented early in the test and thus assumed to be relatively easy were in fact quite difficult for the children in our sample. Examples are item 3 (spoon) and item 8 (cup). At the same time, there were also items that are assumed to be relatively difficult that turned out to be less difficult than some of the earlier presented items. Examples are item 38 (*penguin*) and item 58 (*panda*). Note that these items were assumed to be more difficult by the developers of the PPVT, as indicated by their position in the tests (easy items occur early, harder items later). Overall, the items in the ACCE-V increase monotonously in difficulty, whereas the difficulty of the items in the PPVT does not.

Note that the current version of the ACCE-V does not have a stopping rule, since the test is brief. We re-analyzed the data using a (hypothetical) stopping rule of three incorrect responses in a row and four incorrect responses in a row, respectively. Using the stopping rule did not affect the instrument’s reliability.

Finally, we used the Mantel–Haenszel method (Mantel and Haenszel, 1959) for Differential Item Functioning (DIF) to test for each item whether it was more difficult for either boys or girls. Three items in Form A were potential DIF items. Two showed slight advantages for girls and one an advantage for boys: *grandmother* (65% correct among girls, 56.6% correct among boys), *butterfly* (68.1% correct responses among girls, 56.8% correct responses among boys), and *triangle* (41.1% correct responses among girls, 49.2% correct responses among boys).

### RQ2: Assessment of Chinese Children’s English, Children’s Demographic and English Learning Experiences

**FIGURE 7:**
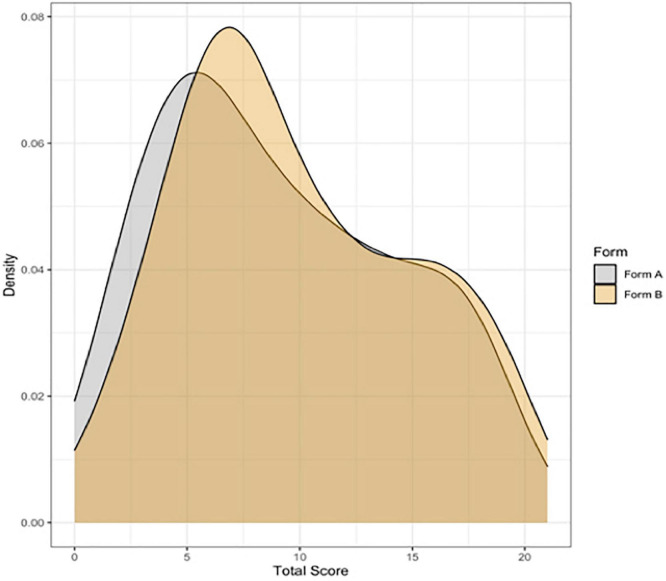
Density plots showing the distribution of scores for the ACCE-V Form B. *N* = 1724 (877 for Form A, 875 for Form B).

The overall mean score for Form A was 9.09 points (range: 0–20 points, *SD* = 5.25), and 9.78 points for Form B (range: 0–20 points, *SD* = 5.12). Figure 7 shows the distribution of scores for both forms.

The ACCE is not designed to provide any age or grade norms, as quality and quantity of English language instruction vary widely between different preschools, and some children may have started English instruction at a later age than others. However, to provide an overview of children’s performance across different ages in the sample, Table 7 shows the measures of central tendency for each 6-months age band.

**TABLE 7:** Number of children, mean score, median score, and standard deviation for 6-month age bands.

	ACCE-V Form A				ACCE-V Form B			
	*N*	Mean	Median	*SD*	*N*	Mean	Median	*SD*
3.0–3.5	36	7.78	7	4.30	36	8.08	8	4.21
3.6–3.11	109	7.83	7	4.60	109	8.61	8	4.66
4.0–4.5	107	9.00	9	4.96	107	9.82	9	4.96
4.6–4.11	124	9.28	8	5.27	124	10.25	9	4.97
5.0–5.5	152	10.33	10	5.51	152	11.10	11	5.13
5.6–511	165	9.14	8	5.31	163	9.55	8	5.16
6.0–6.5	119	8.49	7	5.20	119	9.21	7	5.16
6.6–6.11	65	6.76	6	4.70	65	7.38	6	4.63

In addition to reporting by age group, we used regression models to observe how children’s demographic characteristics (gender, age) and English learning experience (whether there are foreign teachers in kindergartens, kindergarten tuitions) predict children’s English vocabulary skills.

In Form A, girls scored an average of 9.36 points (range: 0–20, *SD* = 5.28), and boys scored an average of 8.88 points (range: 0–20 points, *SD* = 5.23). In Form B, girls scored an average of 10.01 points (range: 0–20, *SD* = 5.13), and boys an average of 9.6 points (range: 0–20, *SD* = 5.10). We used a Bayesian *t*-test from the BayesFactor package (Morey et al., 2015) to test if either gender performed better than the other. A traditional *t*-test can determine if there are significant differences between the two groups. However, a non-significant result does not prove that there is no difference, as a non-significant result can also be due to insufficient data (Dienes, 2014). In contrast, a Bayesian *t*-test allows quantifying the evidence for or against there being a difference between two groups. The Bayes factor (i.e., the ratio of the likelihood of there being a difference between girls and boys to the likelihood of there not being a difference) was 0.19 for Form A and 0.15 for Form B, which is both considered “substantial evidence” (Wetzels et al., 2011) for the absence of a gender difference.

To test the assumptions about the data, we estimated the variance inflation factor (VIF) to check for multicollinearity, a normal predicted probability plot to check for normality, and a residuals plot to evaluate the homoscedasticity of errors. Both tuition and foreign teacher showed signs of multicollinearity. However, we decided to retain these two variables in our models for two reasons. First, tuition and foreign teacher were theoretically important to our model and were potentially able to explain variance on different levels (classroom level and school level). Second, multicollinearity inflate the variance and Type II error, but as will be shown below, Type II errors did not occur (as there were no null results). The assumptions of normality and homoscedasticity were both met.

Table 8 showed the results of multiple regression models. When controlling for foreign teacher, gender, housing price, and tuition, a month increase in children’s age-predicted score increase of 0.29 and 0.28 standard deviations change in Form A score and Form B score, respectively (both *p* < 0.001). When controlling for foreign teacher, children’s age, housing price, and tuition, girls performed 0.06 and 0.05 standard deviations better than boys in Form A and Form B, respectively (*p* < 0.05). It is worth noting that when controlling for age, gender, housing price, and tuition, foreign teacher predicted an increased score of 1.12 and 1.08 standard deviations in Form A score and Form B score (*p* < 0.001). This means that the effect of having a foreign teacher is much larger than the effect of age (0.29 and 0.28 standard deviations).

**TABLE 8:** Results of multiple linear regression models predicting the ACCE-V scores.

	ACCE-V Form A			ACCE-V Form B		
	*b*	Standard error	β	*b*	Standard error	β
Intercept	–4.81	0.90		–3.40	0.88	
Children’s age (in month)	0.13***	0.01	0.29***	0.12***	0.01	0.28***
Foreign teacher	13.68***	1.25	1.12***	12.79***	1.23	1.08***
Gender (girl)	0.64*	0.25	0.06*	0.53*	0.25	0.05*
Housing price	0.06***	0.01	0.33*	0.06***	0.01	0.32***
Tuition	–0.0005***	0.00	–0.58***	–0.0005***	0.00	–0.52***

*p < 0.05; ***p < 0.001.

As we hypothesized earlier, although significant, a child’s age was not the most important predictor of English vocabulary knowledge. More important than age was the English learning environment for children. Children who study in classes with foreign teachers had significantly higher scores than children without foreign teachers (Form A: β = 13.68, *p* < 0.001; Form B: β = 12.79, *p* < 0.001).

## Discussion

Despite the growing demand for English instruction for young children in China, educators and researchers do not have the right tools that allow them to assess children’s vocabulary knowledge. Existing tests like the PPVT and the BPVS are not suitable, because they were developed for first language learners. This is reflected both in the selection of items, which is based on the age of acquisition in an English-speaking environment, and in the depiction of items, which is in accordance with American and British cultural norms.

Our goal with the ACCE-V was to develop an English vocabulary test specifically for young Chinese learners of English. Drawn from textbooks used in Chinese elementary schools, the items are selected to capture school-relevant vocabulary rather than vocabulary that a child growing up in an English-speaking, Western environment would acquire through daily interactions. By using Chinese cultural visual conventions in the drawings, the ACCE-V improves children’s chances of recognizing the intended meaning of a drawing. The multiple-choice format is a familiar format for Chinese children, and the test is easy to administer. With only 5–10 min, the ACCE-V is a short test and can be used in combination with other tests.

The authors and the PACE Research Institute will be responsible for holding and distributing the test. We expect two groups interested in this test, researchers and schools. Eligible researchers from universities or research institutions may contact the authors to request a test, and PACE Research Institution is responsible for training investigators who will administer the tests. Schools or school districts that wish to use ACCE-V can contact PACE, and PACE will arrange for trained personnel to administer the tests.

Two field studies with a combined sample size of more than 1,000 children showed that the ACCE-V has very good psychometric properties with respect to alternate form reliability, test–retest reliability, and internal consistency. IRT analyses indicate the range of difficulty of the items is appropriate for the target population, and that the items are good at discriminating between children with lower ability and those with higher ability.

The test scores showed high correlations with the PPVT-4. Both the ACCE-V and the PPVT are intended to measure children’s English receptive vocabulary, so a high correlation is expected and desirable as an indicator of concurrent (criterion) validity. However, as the analysis of the difficulty indices of items in the PPVT showed, some items that are supposed to be relatively easy for L1 speakers were difficult for our L2 learners, so the assumed progression in difficulty in the PPVT does not necessarily hold for L2 learners.

In our view, observations like these make the case for dedicated L2 vocabulary assessments like the ACCE-V. It is important to note that this is not a critique of the PPVT and similar tests as they are intended—as assessments for L1 learners. Researchers and educators tend to use instruments meant for L1 learners, because they are widely used and because they have been psychometrically validated. However, the validation pertains to the target population (L1 learners), and the tests should not unquestioningly be assumed to be suitable for other populations. While the ACCE-V’s concurrent validity has been demonstrated, we are planning to collect data on children’s English grades in primary school to evaluate the ACCE-V’s predictive validity.

The development of the ACCE-V receptive vocabulary test is part of a larger effort to develop culturally appropriate assessments for young EFL learners. This is not just a challenge for Chinese young English learners.

We see our effort related to the Global English Language Teaching (GELT) framework (Rose and Galloway, 2019). The framework problematizes the conception of English as primarily the language of “Inner Circle” countries (Kachru, 1992), and not the globalized language it is, with many different contexts, uses, and users. Within the conventional approach to EFL teaching and testing, the ultimate goal of learning English is to achieve native-like proficiency, with native-likeness usually being defined by the (idealized) standards of American and British English. The target audience in this approach are also native speakers. Along with this goal often also comes the superimposition of certain norms of the native English-speaking cultures in teaching materials and language tests. In contrast, approaches like GELT assume that the goals for learning English can be manifold—in the case of young EFL learners, it is usually being able to follow and participate in English classes in elementary and middle school, with the longer-term goal of communicating with both native- and non-native speakers, and consuming and producing content from their own culture and from other cultures.

Moving toward a GELT approach in teaching and assessment in the early years is not a call to abandon any inclusion of culture from countries like Britain, Australia, or the United States. Rather, the appeal is to focus more on what is relevant to young L2 learners in their immediate environment and to become aware of and reduce the biases inherent to the traditional approaches. Tests and assessments are obviously only one aspect in the larger system of foreign language learning. However, there is general consensus that testing has an influence on teaching and learning, an effect termed ‘washback’ (Alderson and Wall, 1993). Tests and examinations have a long tradition in China, and the Chinese educational system is geared toward tests and assessments, including English (Cheng and Curtis, 2009). With the growing demand for early English instruction and the current lack of evaluation criteria, new assessments in that domain are likely to influence teacher and parent education decisions. Introducing culturally appropriate language assessments may therefore be a useful way to initiate changes in early English language teaching. Thus, while the primary goal of the ACCE-V is to provide educators and researchers with a valid, culturally appropriate instrument to measure young Chinese children’s English vocabulary, we hope that developments like this one can also have a positive influence on English teaching and testing culture in China in general.

## Limitations

We recruited more than 1,000 children for this study, and all came from the two most economically developed cities in China. Therefore, the conclusions of this study should be very cautious when extending to children learning English in other areas, especially the rural areas in China. In addition, our research data is cross-sectional, and we did not collect longitudinal data on children; thus, we cannot discuss whether ACCE-V can capture the development of children’s long-term English proficiency. In the following research, we will expand our sample, recruit children from other cities, and track the English development of these children longitudinally.

We note that a few children (less than 3% of the total) achieved maximum scores, indicating the possibility of a ceiling effect. We plan to expand the current version of the ACCE-V to include more difficult items. Depending on the resulting length, future versions of the ACCE-V may include a stopping rule.

## Conclusion

The goal of the ACCE-V is to provide educators and researchers with a valid, culturally appropriate instrument to measure young Chinese children’s English vocabulary. In this study, we documented the design process of the ACCE-V and demonstrated its reliability and validity. We showed that the ACCE-V has good psychometrically properties. The authors and the PACE Research Institute plan to open the access of ACCE-V to qualified educators and researchers (e.g., certificated practitioners of English education institutions, researchers with sufficient educational psychology training) and provide them with ACCE-V related training. Before using the ACCE-V, the tester must pass the exam of the ACCE-V design team. As an alternative vocabulary test for young English learners in China, we will use ACCE-V to answer research questions related to Chinese children’s English development, such as the relationship with Chinese proficiency, family socioeconomic status, and family literacy environment.

## Data Availability Statement

The datasets presented in this article are not readily available because the data that support the findings of this study are available from Pace Research Institute but restrictions apply to the availability of these data, which were used under license for the current study, and so are not publicly available. Data are, however, available from the authors upon reasonable request and with permission of Pace Research Institute. Requests to access the datasets should be directed to PW, lukewpz@gmail.com.

## Ethics Statement

The studies involving human participants were reviewed and approved by Pace Research Institute. Written informed consent to participate in this study was provided by the participants’ legal guardian/next of kin.

## Author Contributions

LR: conceptualization, methodology, formal analysis, writing—original draft, and visualization. PW: resources, data curation, methodology, formal analysis, project administration, and visualization. SC: conceptualization, methodology, formal analysis, supervision, writing—review and editing, project administration, and funding acquisition. All authors contributed to the article and approved the submitted version.

## Conflict of Interest

The PACE Research Institute is applying for a patent in China for the ACCE-V and intends to use the test commercially. The authors have received consulting fees and travel grants from the PACE Research Institute and would receive royalties from the ACCE-V should the patent be granted, and the test be used commercially.

## Publisher’s Note

All claims expressed in this article are solely those of the authors and do not necessarily represent those of their affiliated organizations, or those of the publisher, the editors and the reviewers. Any product that may be evaluated in this article, or claim that may be made by its manufacturer, is not guaranteed or endorsed by the publisher.

## Footnotes

1 Age is reported in the format years;months.

2 The category ‘attributes’ comprises both adjectives and adverbs.

3 The mean concreteness scores for these words have been reported as 2.15 for *difference*, 2 for *again*, 5 for *banana*, and 3.75 for *running* (Brysbaert et al., 2014).
